# Collaboration of primary care and public health at the local level: observational descriptive study of French local health contracts

**DOI:** 10.1017/S1463423620000559

**Published:** 2020-12-14

**Authors:** Candan Kendir, Eric Breton, Yann Le Bodo, Yann Bourgueil

**Affiliations:** 1 Mission RESPIRE (EHESP-CNAMTS-IRDES – EA MOS 7348 EHESP), 20 avenue George Sand, 93210 la Plaine, Saint Denis, France; 2 Social and Human Sciences Department, École des hautes études en santé publique, 15 Avenue du Professeur Léon Bernard, 35043 Rennes, France; 3 Social and Human Sciences Department, Laboratoire Arènes (UMR CNRS 6051), 35700 Rennes, France

**Keywords:** community care, primary healthcare, primary care, public health

## Abstract

**Aim:**

In this paper, we report on a study investigating the involvement of primary care providers in French local health contracts.

**Background:**

Worldwide actions are carried out to improve collaboration between primary care and public health to strengthen primary healthcare and consequently community health. In France, the local health contract is an instrument mobilising local stakeholders from different sectors to join in their actions to improve the health of the population.

**Methods:**

We developed an instrument to analyse the frequency and nature of involvement of primary care providers in 428 action plans extracted from a sample of 17 contracts (one per French region). The number of primary care actions were counted, and thematic analyses were conducted to identify the nature and level of involvement of the professionals.

**Findings:**

Primary care providers were involved in 20.1% (*n* = 86) of the action plans and were mostly described as a target of the action rather than leaders or partners. Within those action plans, 76.7% (*n* = 66) of these action plans aimed to improve access to care for local communities; an issue that appears as the main driver of collaboration between public health and primary care actors.

## Introduction

Primary healthcare is considered as one of the most effective ways to promote the highest possible health standards for all by emphasising universal health coverage. The WHO defines primary healthcare as ‘*a whole-of-society approach to health that aims equitably to maximize the level and distribution of health and well-being […] along the continuum from health promotion and disease prevention to treatment, rehabilitation and palliative care…’* (WHO Regional Office for Europe, 2019).

Collaboration of primary care and essential public health functions is considered to be one of the building blocks of the primary health care approach together with people’s and communities’ empowerment and multisector action (WHO, 2018). Primary care services are clinical care services delivered on an individual basis that are accessible for everyone, dealing with the largest share of health complaints by a comprehensive approach in a continuous manner, and coordinated between levels of care (Starfield *et al.*, 2005). Public health for its part is often defined as ‘the art and science of preventing disease, prolonging life and promoting health through the organized efforts of society’ (Winslow, 1920). Public health intervention is carried out at the population level. Notwithstanding the important differences between the two fields of practice, a collaboration between public health actors and primary care providers is often impeded by organisational or communication issues between professionals, such as problems in sharing data or developing a common vision for the community (Lebrun *et al.*, 2012; Pratt *et al.*, 2018).

In recent years, some countries (Committee on Integrating Primary Care and Public Health; Board on Population Health and Public Health Practice; Institute of Medicine, 2012; Booth *et al.*, 2016; Gosling *et al.*, 2016; Lionis *et al.*, 2018) moved forward and created new local networks to integrate primary care and public health. However, accounts of these experiences are still sparse, evidence on health impacts is limited and there is a lack of literature on how to foster the development of these collaborations (Allen *et al.*, 2018). In countries with a strong primary care system, such as the Netherlands and England, linkages and articulation between primary care and public health are limited (Hone *et al.*, 2018), and hamper the efficacy of primary healthcare.

The French primary care system is a ‘medium level strength’ system with a long history of professional non-hierarchic primary care model (Kringos *et al.*, 2013). Since 2005, different reforms have been implemented to support the development of teamwork, skill mix and case management in primary care (Bourgueil *et al.*, 2009, 2008). Contiguously, public health policies have been decentralised with, in 2009, the creation of Regional Health Agencies (*LOI n° 2009-879 du 21 juillet 2009 portant réforme de l’hôpital et relative aux patients, à la santé et aux territoires*, 2009). Amongst other mandates, Regional Health Agencies are responsible for the implementation and adaptation of national health strategies through regional health programs. To this end, one of the instruments that are promoted is the local health contract. These contracts fostering local public health actions are co-signed by the Regional Health Agencies and local governments such as municipalities, syndicate of towns, or other subregional entities with contributions from other local partners such as local branches of state agencies. In local health contracts are defined priorities and a set of actions to be carried out for the next one–five years by aligning national health priorities with regional and local ones. It covers actions in health promotion, disease prevention and access to health services, with an overarching aim to reduce social and territorial health inequalities (*LOI n° 2009-879 du 21 juillet 2009 portant réforme de l’hôpital et relative aux patients, à la santé et aux territoires*, 2009).

Local health contracts intend to push local stakeholders from different sectors to join in their actions through local partnerships and engagement of the community in order to improve environmental and social contexts, to increase access to care, foster disease prevention and health-promoting actions (Haschar-Noé and Salaméro, 2016). These public health actions involve a diverse set of stakeholders (local elected officials, social services, health-related networks such as diabetes network, patient associations, hospitals, health professionals and also community-based associations and more).

Although no permanent funding is specifically devoted to local health contracts, Regional Health Agencies often fund the conduct of the diagnosis on which is based the identification of priorities and may share the cost of the coordination (full-time or part-time position) with the local authority. In addition, the signature of a contract comes with some benefits for the local partners. For instance, it creates an opportunity for local elected officials to claim legitimacy to intervene on health and display to their electorate their leadership on health and health-related (Salaméro and Haschar-Noé, 2017). For the above-mentioned local stakeholders relying on public subsidies to maintain their staff and activity, being involved in a local health contract may increase the likelihood of funding attribution through grant applications to the Regional Health Agencies (Haschar-Noé *et al.*, 2015; Honta *et al.*, 2018). For other partners such as gyms, it is a way to promote their business plan such as by agreeing on providing special services to individuals oriented by primary care providers or by organising community activities to promote healthy lifestyles. Some authors suggest that the more concrete, aligned with regional priorities, and supported by local actors’ local plans are, the more likely they will attract funding from Regional Health Agencies (Haschar-Noé *et al.*, 2015; Salaméro and Haschar-Noé, 2017).

Local health contracts’ context may offer health care providers in primary care settings an opportunity to lead or get involved in public health actions. These actions could consist of an effective orientation to health promotion services for a better use of resources by individuals or for the involvement of primary care professionals in the community’s activities such as attending the sports festivals and discussing the physical activity. Therefore, theoretically, local health contracts may act as an instrument that potentially enhances primary healthcare and improves the health and well-being of individuals and the population. Today, as 10 years have passed since the introduction of the law defining the mission of primary care providers, creating Regional Health Agencies and introducing local health contracts, over 400 contracts were signed by local-level partners across the mainland and overseas French territories (Breton and Team CLoterreS, 2020).

One important feature of local health contracts is their flexibility in terms of the nature of the problems addressed and stakeholders involved. This is likely to generate variability in directions when comes the time to prioritise issues, to define respective roles and responsibilities and to articulate local health contracts with other instruments of governance on a given territory (Jabot and Laurent, 2018).

At the crossroads of primary care and public health, the primary health care approach seeks to extend the scope of primary care services to meet public health goals at the meso level in order to improve community health (Levesque *et al.*, 2013). The meso level shows how professionals, civil society organisations and communities can interact to strengthen the ‘primary care’ that delivers care at the micro level (De Maeseneer *et al.*, 2014). We postulate that local health contracts can be potentially designed as instruments to foster primary healthcare at the meso level. This could facilitate linkages between health professionals and public health ones together around the community’s needs.

Overall, the expected structuration of integrated professional community-based care sets a context increasing the likeliness of collaboration between primary care and public health at the local level (Ministère des Solidarités et de la Santé, 2019a), but the extent to which local health contracts may stand as instruments for this convergence remains under-documented (Breton and Team CLoterreS, 2020).

In this context, our objective was to investigate the involvement of primary care professionals in local health contracts, more specifically, we examined (1) whether and how frequently primary care actors were involved in local health contracts; (2) how primary care professionals were involved; and (3) in what type of public health actions primary care actors collaborated with local partners.

## Methods

This exploratory study was conducted as an offspring of the CLoterreS research project. For this project, with the guidance of international guidelines, the CLoterreS team has developed a database of local health contracts. For each contract, the database includes information, for example, on the main health issues to be addressed on the territory, on the affiliations of the signatories of the contract, on the themes addressed in each action plan, be these related to the social determinants of health, health promotion activities, disease prevention activities and health care services.

An observational documentary analysis on the signed contracts was conducted between January 2018 and June 2018. This study is based on a stratified sample of 17 local health contracts (one per French region) randomly selected from the population of 165 contracts signed between January 2015 and March 2018 inventoried through the CLoterreS research project (Table 1). Although the first local health contract dates back from 2011, the CLoterreS team selected this period to reflect current practices and better inform decisions. To be eligible, the full contract (terms and action plan) had to be available in the CLoterreS database in November 2018. Considering the variety of regional policies and territories across the country, this strategy proved instrumental in providing a diversity of cases (Table 2) rather than a representative sample of all local health contracts.

**Table 1.** National census of local health contracts (source: CLoterreS project)

French regions^a^	Total number^b^ of local health contracts signed between January 2015 and March 2018
Auvergne-Rhône-Alpes	12
Bourgogne-Franche-Comté	15
Bretagne	10
Centre-Val de Loire	17
Corse	3
Grand Est	12
Guadeloupe	4
Guyane	2
Hauts-de-France	7
Ⓘle-de-France	30
Martinique	2
Normandie	5
Nouvelle-Aquitaine	15
Occitanie	16
Océan Indien	3
Provence-Alpes-Côte d’Azur	2
Pays de la Loire	10
**TOTAL**	**165**

a Names corresponding to the 17 Regional Health Agencies in 2018.

**Table 2.** General characteristics of the sample of 17 local health contracts included in the study (source: CLoterreS project)

# Local health contact^a^	Year of signature	Category of population size on the territory^b^	Town (T), Syndicate of municipalities (S), Other (O)	Predominantly rural area^c^	Number of signatories	Main signatories beyond the Regional Health Agencies and the local government^d^	Number of action plans	Main areas addressed in the action plan
1	2017	4	T	No	15	Regional government, subregional government, other local government, prefect, social health insurance funds, health/long-term care centres, mutual funds	55	Infancy and youth health, women health, primary and secondary prevention of chronic diseases, mental health, healthy living environments, coordinated healthcare, access to rights and care for disadvantaged groups
2	2016	2	T	No	4	Other local governments, prefect	53	Access to primary care, environmental health, prevention of chronic diseases, health promotion, health of the elderly and disabled persons, addictions
3	2015	2	O	Yes	7	Regional government, prefect, health/long-term care centres, education authorities	19	Prevention and health promotion across the life course, socio-demographic evolutions of the territory (access to care, isolation, family caregivers), communication, coordination and evaluation
4	2018	3	S	No	7	Subregional government, prefect, social health insurance funds, health long-term care centres	8	Access to primary and emergency care, health of the elderly, environmental health, mental health, maternal and infant health, youth health, access to rights, prevention and care for disadvantaged groups
5	2016	2	T	No	4	Prefect	56	Needs analysis and coordination of actions, healthy living environments, mental health, nutrition, prevention of risky behaviours, prevention of chronic diseases, access to care
6	2015	2	O	Yes	14	Subregional government prefect, social health insurance funds, health/long-term care centres	17	Mental health and healthy environments, addictions, prevention of cardiovascular diseases, access to care
7	2017	1	T	No	4	Prefect, social health insurance funds	14	Prevention of risky behaviours in youth, screening of diseases, therapeutic patient education, emergency care services and access to care
8	2018	1	O	Yes	10	Regional government, prefect, social health insurance funds	23	Quality of life on the territory, coordinated healthcare and telemedicine, health and well-being for youth, elderly people, disabled persons, deprived groups
9	2015	2	S	Yes	2	-	18	Coordination of healthcare and access to care, access to rights and coordinated health services for vulnerable groups, prevention and health promotion involving professionals and inhabitants
10	2015	2	T	No	4	Prefect, social health insurance funds	27	Prevention of chronic diseases, infancy and youth health, mental health, health of the elderly, organisation of primary care, inclusion of health matters in other policy areas (*Politique de la ville*)
11	2016	4	T	No	4	Prefect	21	Health promotion and education, prevention of infectious diseases, addictions, long-term care, social inclusion of disabled persons, mental health, access to care for disadvantaged groups
12	2015	2	O	Yes	3	Prefect	15	Access to care, coordination of primary care, therapeutic patient education, prevention and screening of diseases, long-term care
13	2015	1	O	No	2	-	5	Nutrition and obesity, vector-borne diseases, mental health, health surveillance, coordinated healthcare
14	2015	3	T	No	3	Prefect	10	Access to rights and care for disadvantaged groups, healthy living environments, prevention of chronic diseases, addictions, mental health, sexual and reproductive health
15	2015	2	S	No	9	Prefect, social health insurance funds, health/long-term care centres	34	Access to rights and care for disadvantaged groups, youth health, health of the elderly, addictions, mental health
16	2016	4	S	No	5	Prefect, social health insurance funds	14	Prevention and health promotion, access to rights and care, mental health, health of the elderly, environmental health
17	2015	5	T	No	4	Subregional government, prefect	54	Children and adolescent health, health of the elderly, health of disabled persons, access to rights and care, screening of diseases, addiction, mental health, vaccination

a Random numbering, independent from the numbers associated with the 17 French regions as shown in Table 1.

b Categories (number of inhabitants): (1) < 10 000; (2) 10 000–50 000; (3) 50 000–100 000; (4) 100 000–500 000; (5) > 500 000.

c Territory covered by the local health contract corresponding predominantly (or not) to a rural area (*Pays, PETR, parc naturel*).

d Prefect: (sub-) regional state representative and its services.

## Material analysed

A local health contract takes the form of a document whose structure usually reflects the template suggested by the Ministry of Health (Agence Régionale de Santé, 2018). It covers sections such as context, identification and engagement of partners and a list of action plans. An action plan includes a thematic title, a statement of the general objective, a statement of its specific objectives, a description of the actions, partners who contributed to design the action plan, the actors involved to implement the actions, budget, timeline and evaluation objectives. In practice, due to the local context, the methodological resources and actors’ configuration, there are variations between and within local health contracts. For instance, while a contract may feature 5 action plans, another one may have 50. The period that covers the contract also varies and so the number and characteristic of partners for each action plan.

## Dataset and analysis

A dataset in Excel format was created, and the coding was carried out at the action plan level for each of the 17 contracts. From the 17 contracts, we retrieved 440 action plans. Insufficient information on 12 of them brought the number of plans down to 428.

In order to analyse whether and how frequently primary care professionals were involved in the local health contracts, we first created a nominal variable to identify the presence of primary care professional’s involvement. Two researchers (CK and YB) coded the actions as follows and reached a consensus when disagreeing: the action (1) includes the involvement of a primary care professional or organisation; (0) does not include any primary care professional or organisation; (99) includes a health care professional but primary care professional is not mentioned specifically. Primary care actions were defined by the involvement of professionals or health care organisations, which were identified through a set of keywords (see Appendix 1). As an example of keywords, a multiprofessional primary care practice (*maison de santé pluriprofessionnelle; MSP*) is a grouping of different primary care professionals (general practitioner, nurse, physiotherapist, midwife and others) usually practicing in the same venue but not necessarily. For the qualitative analysis of the data, the actions that involve primary care were retrieved and thematic analysis was performed against the involvement of primary care professionals:

- In order to identify the nature of the involvement of primary care professionals in the local health contracts, we carried out a thematic analysis of each action plan. We followed an inductive approach by allowing iterative revisions during the coding. These responsibilities could range from being a leader or partner in developing the action plan or being the actual target of the action. In order to perform this coding, we reviewed the whole action plans rather than relying only on how such responsibilities were described in a specific box of the plan.
- In order to analyse the nature of public health actions involving primary care professionals in the local health contracts, we used two sources as a starting point (WHO The 10 Essential PublicHealth Operations, 2019). These sources are the WHO Essential Public Health operations and the French law on public health actions for primary care professionals (Code de la santé publique - Article L6323-3). Following these, we adopted a deductive-inductive approach by allowing ourselves to modify our classification in light of the material analysed. At the end of this process, we created our final nominal variables as (1) coordination of care, (2) health protection including environmental occupational, food safety and others, (3) health promotion including actions to address the social determinants of health and health inequity, (4) disease prevention including early detection of illness, (5) patient education programs, (6) access to care. When needed, double coding was applied.

Following the thematic analysis, a descriptive analysis was done at the action plan level (*n* = 428) and at the contract level (*n* = 17) by using Microsoft Excel version 16.02 and SPSS 15.0 software.

## Results

The mean number of action plans per local health contract was 25±17.58 (min-max = 5–55, median = 19). Amongst the 428 action plans analysed, 20.1% (*n* = 86) included at least one component involving primary care providers, 28.5% (*n* = 122) involving some kind of health care providers, that is, primary care professionals not specifically mentioned. In Figure 1, we show the presence of primary care professionals in each local health contract.

i. The nature of primary care professionals’ involvement
  **Figure 1.**
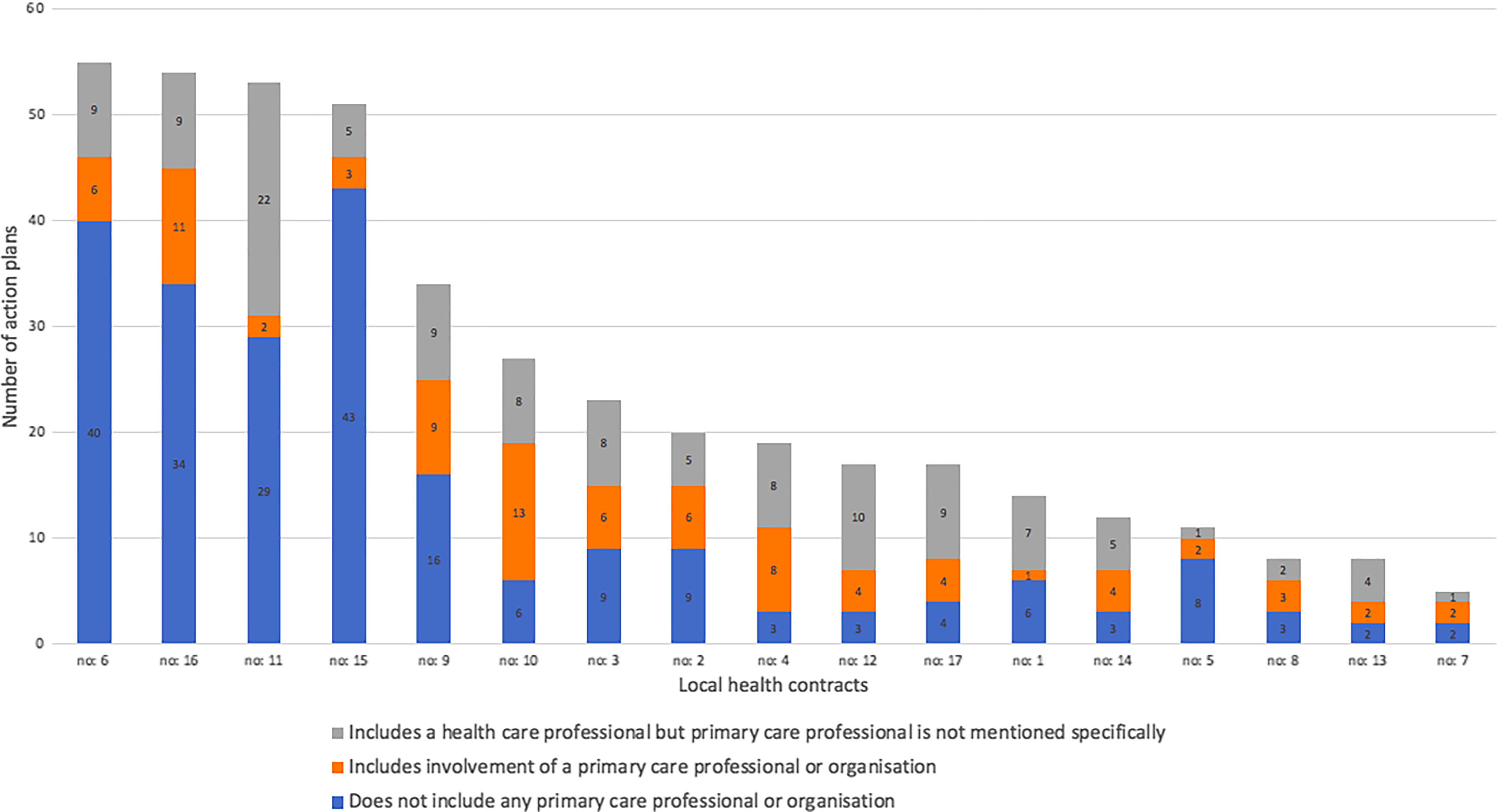
Presence of primary care involvement in the local health contracts.

Amongst all actions that involve specifically primary care professionals or organisations (*n* = 86), 5 actions (6%) were discarded for lack of information on the nature of professional involvement, leaving 81 action plans for the analyses.

a. Primary care professionals are rarely the action leader (9%, *n* = 7)

In very few cases, primary care professionals stood as leaders of the action. When they did, they were either mentioned as being the main person/organisation in charge of the action or, co-heading the development of the action plan and its implementation. Here are two examples:

‘*Objective of the action: Improve access to health care administrative rights for vulnerable population*

*Actions planned:*

- *Provide financial and fundamental support to multiprofessional primary care projects to support their actions in the priority districts for urban policy*
- *Support teams of practicing health professionals in the development and implementation of their health project*

*Leaders of the action:* Regional Health Agencies*, city and an association of health professionals’ (FA. 6.51.)*

‘*Objective of the action: Sustain primary care services by creating health centres*

*Actions planned:*

- *Guarantee access to primary healthcare for the population of [name of territory] through the presence of three multiprofessional primary care practices*
- *Establish multiprofessional primary care teams which will be the foundation of each health centres*
- *Formalize a health project for each health centre, by self-employed health professionals, validated by the Regional Health Agencies*

*Leader of the action: Community, Association of Multiprofessional primary care practice (…)’ (FA.9.11.)*

a. Primary care professionals are sometimes action partner (25%, *n* = 20)

As illustrated in the following extracts of two local health contracts, when the primary care professionals were the partner of the action, they were not stated as leaders of the action, but they had an active role in its development and implementation. In this category, we also identified actions that include primary care organisations as a partner but not necessarily professionals of the region themselves.

‘*Objective of the action: To allow the inhabitants of the territory to have rapid access to an ophthalmological examination within the validated framework of the follow-up of diabetics*

*Actions planned:*

- *Tele-ophthalmology*

*Leader of the action: Ophthalmologists*

*Partners:* Regional Health Agencies*, local branch of social security, association of self-employed health professionals, general practitioners, nurses, health professional associations.’ (FA.3.4.)*

In certain actions, even though primary care professionals were mentioned as a partner of the action, the text did not state their responsibilities.

‘*Objective of the action: To improve vaccine coverage*

*Actions planned:*

- *UC-IRSA (regional social security organisation for prevention and health promotion) proposes to ensure that children are vaccinated during school registration in town hall and design a similar targeting system for active and inactive people who are unemployed, and who do not necessarily consult general practitioner.*
- *The UC-IRSA can travel to the site to organise sessions of vaccination drives as needed.*

*Leader of the action: UC-IRSA*

*Partners:* Regional Health Agencies*, local branch of social security*, *self-employed doctors, nurses, pharmacists, multiprofessional health practices, associations…’ (FA.4.6.)*

a. Primary care professionals are mostly the target of the action (66%, *n* = 54)

For the majority of actions, primary care professionals were the target of the action. That means they did not have any responsibility in leading the action or developing it. They were little more than a channel to reach the target population.

‘*Objective of the action: Fighting sedentary lifestyles as part of primary and secondary prevention, through the implementation of physical activities/ sports adapted to the target groups*

*Action planned:*

- *Creation of a network including family doctors, physical activity coach for people with a chronic disease, coordinator within the local government supporting the project, sports educator of a local sports association. Each protagonist will be associated to the network through a formal agreement. A training component specific to the “Prescription of Sport” project for sports educators is included in the scheme*.

*Leader of the action: City of (…).*

*Partners: not mentioned’ (FA.15.4.10.)*

‘*Objective: To improve familiarity and promote respite care for family carers and elderly people*

*Action planned:*

- *Prioritize the establishment of medical services (health centres or others) in town centres in order to facilitate the accessibility of these services to elderly people whose mobility is reduced*

*Leader of the action: Regional Health Agencies and Departmental Council, co-management with the support of the project team (Gerontology association, Health Cooperation Group).’* (FA.13.3.)

i. Type of actions that involve primary care professionals

**Figure 2.**
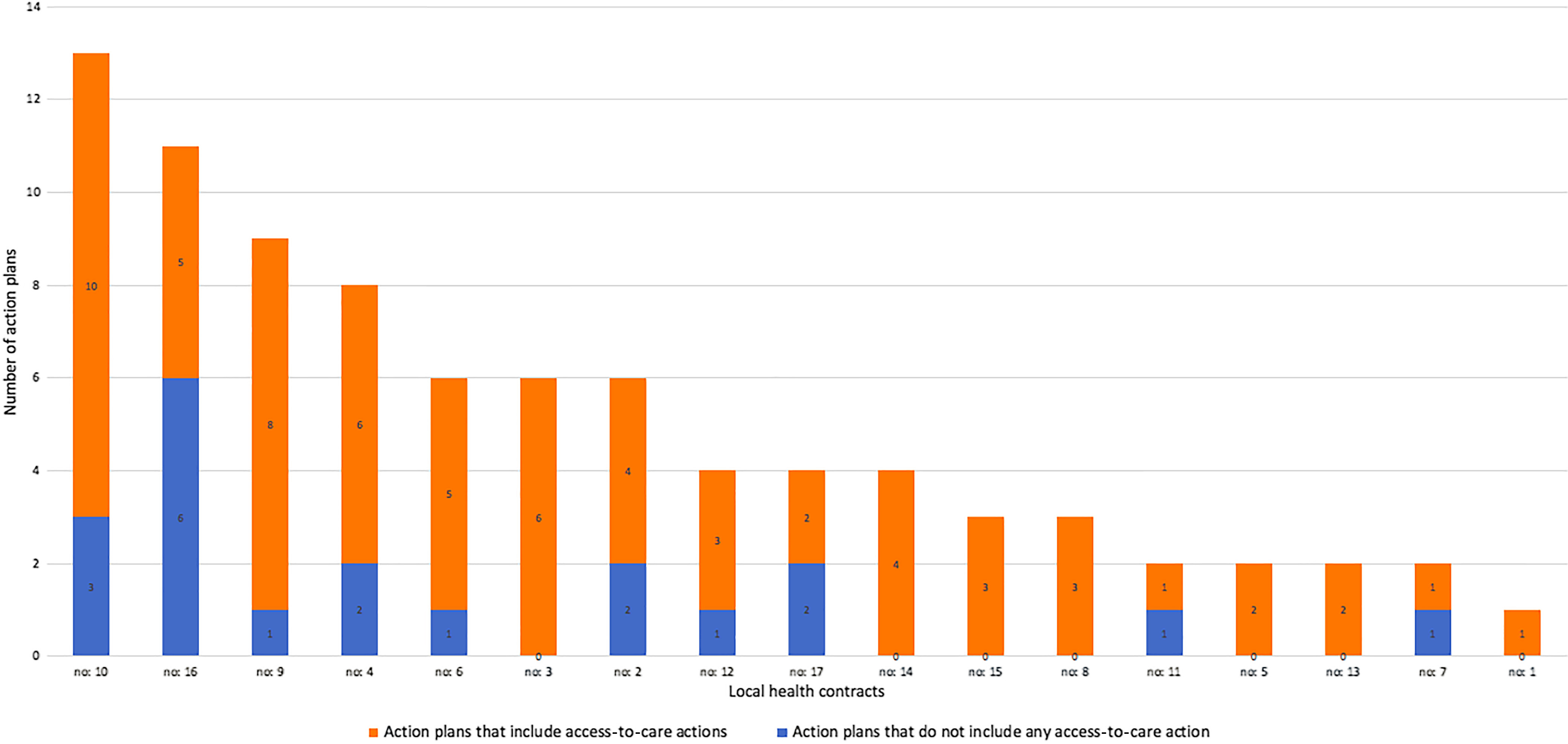
Number of action plans that include (or not) access-to-care actions, amongst all actions which involve primary care professionals.

Amongst the actions involving primary care providers (*n* = 86), 76.7% (*n* = 66) aimed to promote access to healthcare. The breakdown of actions whether they include an access-to-care action for each local health contract is shown in Figure 2.

Amongst access-to-care actions (*n* = 66), 28.8% (*n* = 19) were about access to preventive services, 27.3% (*n* = 18) about coordination to increase access to other care services, particularly to specialised care, 22.7% (*n* = 15) about access to primary care services in general, 15.2% (*n* = 10) about access to health promotive services excluding health education and 6.0% (*n* = 4) were about access to patient education. For example:

‘*Objective of the action: Maintain access to primary care services and continuity of care*

*Actions planned:*

- *Organisation of meetings with general practitioners in order to address the difficulties they face and create a dynamic for a joint project*

*Type of action: Access to care (primary care services) (FA.1.2.1.)’*

‘*Objective of the action: Promote telemedicine projects by facilitating and improving patient pathways with a better coordination between health professionals and allowing habitants to access to health care services*

*Actions planned:*

- *Setting up of a mobile unit for telemedicine services via videoconference in general practice clinics*

*Type of action: Access to care (specialised care services), coordination of care (FA.3.3.)’*

The other actions involving primary care professionals without any access-to-care component (23.3%; *n* = 20) were limited to disease prevention, health promotion, coordination of care and health education. We did not find any health protection-related action involving primary care professionals or organisations such as one addressing environmental health.

‘*Objective of the action: Train care providers and improve communication between providers of home-care services*

*Actions planned:*

- *Train care providers about good use of existing resources in order to improve comprehensiveness and utility of shared information between general practitioners and other home care providers*

*Type of action: Coordination of care (FA.4.10.)’*

‘*Objective of the action: Improve the quality of life of children, adolescents and young adults in the territory*

*Actions planned:*

- *Carry out a half day of exchange with professionals from the neighbourhood around the factors (biological, social, cultural, etc.) that determine a child’s weight gain. Will also be discussed:*
  - ○ *their knowledge and their perceptions in terms of food hygiene*
  - ○ *the needs they identify in this regard in the territory*

*Type of action: Health promotion (FA.6.6.)’*

## Discussion

Our study shows that primary care professionals or organizations have been involved in at least one out of five action plans in the local health contracts studied. Only a few actions were developed under the leadership of a primary care professional or organisation. The majority of actions included primary care professionals or their settings as a means to reach the target population. The majority of actions involving primary care focused on the dimension related to the accessibility of care services, sometimes including public health actions. Besides this, coordination, disease prevention and health promotion actions (without access to care) also included primary care providers, but only marginally.

Many action plans sought to involve primary care professionals. This may be difficult to do in practice, as primary care providers are mostly independent and private providers in France. As the French Government recently set a goal of developing nationally a 1000 communities of health care professionals (Communauté professionnelle territoriale de santé) by 2020, a figure that would represent a twofold increase in multiprofessional primary care practices (Safon, 2018), and comes with the development of coordinative functions and roles, local health contracts could contribute in strengthening integration for the development of these initiatives by allowing increased access to care and prevention (Ministère des Solidarités et de la Santé, 2019b).

The majority of action plans position primary care professionals as a target with very few descriptions of mechanisms to involve them. In some actions, we found the professional associations as partners of the actions. Yet, we do not have information on the nature of the relationship with the practice and involvement of regional health professionals in the project. Several scholars discuss the importance of developing common objectives and a shared vision for a successful collaboration between public health and primary care (Valaitis and Canadian Foundation for Healthcare Improvement, 2013; Pratt *et al.*, 2018, 2017). In a Canadian study accounting for micro-, meso- and macro-level actors of collaboration, it was discussed that such collaboration sharing common objectives should occur at all levels, from top management to practicing professionals (Akhtar-Danesh *et al.*, 2013). This brings to attention the importance of normative function of collaboration such as developing joint missions and the need for developing initial and continuous education regarding collaboration amongst both public health and primary care professionals besides the development of functional enablers.

As regards the type of actions primary care providers are involved in, our analysis points to few linkages between primary care and public health actions in local health contracts. Beyond access to care, the involvement of primary care professionals in public health action plans is rather limited. We found that most action plans involving primary care were oriented towards improving access to care. Additionally, we found actions on the coordination function of primary care, which also increases access to specialist care. Today, a primary health care approach is accepted as a key for universal health access (WHO Regional Office for Europe, 2019). Indeed, accessibility is one of the biggest contributions of primary care to population health improvement (Starfield *et al.*, 2005). This highlights the key role local level governance play in making universal health coverage effective even when it is written in the law of the land (Bourgueil, 2018). Accessibility remains a topical issue in France and elsewhere. This is an issue fuelled by a shortage of medical doctors and also the inadequacy of health care organisation to the changing health needs at the local level (DREES, 2012; WHO, 2016).

We found no action on the social determinants of health or health protection that involve primary care professionals or organisations. Although it is not explicitly one of the missions of the primary care professionals in France, we believe this could also either be explained by our small sample size or the lack of details about the nature of health care professionals mentioned. Indeed, the significant role of primary care in prevention is supported in the literature (Green *et al.*, 2012). We see more and more active involvement of primary care professionals in the health promotion and health protection areas such as environmental health issues (WONCA Working Party on the environment *et al.*, 2019).

The number of instances of participation of primary care professionals or organisations was not linked to the type of actions but more to the local health contract itself. We noticed that there are clusters of actions that involve primary care professionals more frequently in some territories than others. This probably reflects the existing relations between local public health partners and primary care providers in certain local areas. This could be interpreted as a contribution of local health contracts as a means for further integrative instruments.

Finally, one should note that the local health contracts analysed stand as projects whose implementation is planned over a few years. This raises the question of their operationalisation and sustainability in the long term. Additionally, the issue of resource limitation should be acknowledged. As there is no specific budget attached to local health contracts’ implementation, funding decisions by Regional Health Agencies (and evolving national and regional priorities) are likely to impact decisions on which action plans get the priority at the local level. Thus, the leadership of the Regional Health Agencies, which are under national control, should be accounted for.

## Strengths and limitations

This is the first study investigating the involvement of primary care professionals in local health contracts. Thus, it is also the first attempt to identify the typology of actions that involve primary care in the local health contracts in the context of France.

Yet, the study has a number of limitations. First of all, we had a small sample size of local health contracts due to time constraints. However, based on a clustered random sampling method, our sample is diverse, *that is,* each local health contract was retrieved from a different French region thus providing an overview of the national landscape.

Another limitation was the identification of primary care actions in order to create the study dataset. As the contracts were from different French regions, the glossary of terms was not stabilised. In order to standardise, we defined keywords *a priori* to classify the primary care actions (Appendix 1). We also only coded actions when they clearly referred to primary care professionals, organisations or associations. But, by adopting this approach, we probably limited the number of actions considered in the analysis. We likely underestimated the frequency of primary care involvement, which may be between 20 and 48% of actions if we assume that the health care professionals that are not specifically mentioned as primary care professionals could include primary care.

Lastly, the material of the study was made of documents describing actions planned, not actual actions implemented. Additionally, the degree of precision of each action plan varied significantly. As a salient example, in one contract, an action plan was detailed in five pages whereas, in another one, several action plans with different themes were combined in one page. With no information on the actual implementation of the plans, there is a possibility that some may never see the light of the day. Still, our study provides valuable indications on the involvement of primary care professionals and organisations in public health actions at the local level, which is an important first step towards primary healthcare.

## Conclusion

French local health contracts involve primary care providers in at least one-fifth of action plans, mostly based on their accessibility to care and coordination functions, not only at the individual level but also within population-centred action plans.

There is obviously room to further develop community-oriented actions of primary care at the local level. This could be taken into account in order to reinforce local health contracts as a promising primary health care strategy in the context of actual policy with the current development of integrated professional community-based care.

One way to improve the collaboration between primary care and public health could be strengthening the role of primary care as a partner of the public health actions. This includes the development of a joint vision and of missions, goals and objectives amongst different actors at the local level. Much has to be done to operationalise closer collaboration such as improving training and continuous education for health professionals and creating specific positions for public health professionals in the management of projects. Hence, more empirical work is needed to identify cases of successful collaboration at the local level and their contributing factors. Due to the strong policy will to implement rapid changes, interventional research and action research may be an appropriate strategy.

## Appendix 1. Keywords for primary care providers or organisations

- « Soins primaires » Primary care;
- « Soins de premier recours » Primary care;
- « Soins de première ligne » Primary care;
- « Soins de proximité » Community-based care;
- « Médecin généraliste » General practitioner;
- « Médecin traitant » Family doctor/ Family physician;
- « Infirmières » Nurse;
- « Sage-femme » Midwife;
- « Pharmacien » Pharmacist;
- « Laboratoire » Laboratorian;
- « Dentiste » Dentist;
- « Maison de santé pluriprofessionelle » Multiprofessional health practices;
- « Maison de santé » Multiprofessional health centres;
- « Pôle de santé » Health centres;
- « Maison de santé pluriprofessionelle universitaire » Universitary Multiprofessional health practices;
- « SAMU » Emergency health care services.

## Appendix 2. Definition of public health actions

**Table:**

1. Coordination of care	Strategies on the collaboration of care and healthcare between medical and social services and within them.
	For example, care pathway, creation of partnership on health; make professional knows each other to ease the coordination, coordination of patient pathway between different healthcare and care services.
2. Health protection	Strategies on environmental health, occupational health and food safety.
3. Health promotion	Strategies on improving the health and well-being of the population, addressing social determinants of health and health inequalities.
	For example, health education activities, lifestyle changes, sexual health, mental health, tobacco, alcohol, nutrition.
4. Disease prevention	Strategies on prevention of diseases through different levels of actions such as primary, secondary and tertiary.
	For example, vaccination, screening, therapeutic patient education of chronic diseases, rehabilitation.
5. Patient education	Strategies on therapeutic patient education on chronic conditions such as diabetes, hypertension, cancer.
6. Access to care	Strategies that aim to increase access to care/health services directly or indirectly.

## References

[ref1] Agence Régionale de Santé, Guide Methodologique d’élaboration d’un CLS, [Methodological Guide for the elaboration of a CLS]. Retrieved 18 May 2019 from https://www.labo-cites.org/Datas/DT_sant/CLS_TYPE.pdf. [in French]

[ref2] Akhtar-Danesh N , Valaitis R , O’Mara L , Austin P and Munroe V (2013) Viewpoints about collaboration between primary care and public health in Canada. BMC Health Services Research 13, 311 10.1186/1472-6963-13-311. 23945461 PMC3765372

[ref3] Allen LN , Barkley S , De Maeseneer J , van Weel C , Kluge H , de Wit N and Greenhalgh T (2018) Unfulfilled potential of primary care in Europe. British Medical Journal 363, k4469. 30355571 10.1136/bmj.k4469

[ref4] Booth M , Hill G , Moore MJ , Dalla D , Moore MG and Messenger A (2016) The new Australian Primary Health Networks: how will they integrate public health and primary care? Public Health Research and Practice 26, e2611603 10.17061/phrp2611603. 26863166

[ref5] Bourgueil Y (2018) L’égal accès aux soins de qualité pour tous. Un principe constitutionnel fondateur de l’assurance maladie, une valeur professionnelle à concrétiser. [Equal access to quality care for all. A founding constitutional principle of health insurance, a professional value to be put into practice.] Actualité et dossier en santé publique 107, 18–21. [in French]

[ref6] Bourgueil Y , Clément M , Couralet P , Mousquès J and Pierre A (2009) An exploratory evaluation of multidisciplinary primary care group practices in Franche-Comté and Bourgogne. Issues in health Economics No 147.

[ref7] Bourgueil Y , Fur PL , Mousquès J and Yilmaz E (2008) GPs teamed up with nurses: a skill mix experiment improves management of type 2 diabetes patients - Main results of the ASALEE experiment 8. Issues in health economics n:136.

[ref8] Breton E and Team CLoterreS (2020) Contrats locaux de santé [Local health contracts]. Retrieved from https://www.cloterres.fr/travaux-resultats/ [in French]

[ref9] Committee on Integrating Primary Care and Public Health; Board on Population Health and Public Health Practice; Institute of Medicine (2012) Primary Care and Public Health: Exploring Integration to Improve Population Health. *Washington: The National Academies of Sciences*. 24851288

[ref10] Code de la santé publique - Article L6323-3. [Code of public health]. Retrieved 2 May 2017 from https://www.legifrance.gouv.fr/affichCodeArticle.do?idArticle=LEGIARTI000038886477&cidTexte=LEGITEXT000006072665&dateTexte=20190727. [in French]

[ref11] De Maeseneer J , Aertgeerts B , Remmen R and Devroey D (2014) Together We Change. Soins de santé de première ligne: maintenant plus que jamais!, [Primary Health Care: Now More Than Ever]. Retrieved from http://www.hapraktijkvoorbeelden.be/doc/together-we-change-fr.pdf [in French]

[ref12] DREES (2012) *Number of doctors and nurses* Retrieved 17 May 2019 from http://www.data.drees.sante.gouv.fr/ReportFolders/reportFolders.aspx

[ref13] Gosling R , Davies SM and Hussey JA (2016) How integrating primary care and public health could improve population health outcomes: a view from Liverpool, UK. Public Health Research and Practice 26, e2611602 10.17061/phrp2611602. 26863165

[ref14] Green LW , Brancati FL , Albright A and Primary Prevention of Diabetes Working Group (2012) Primary prevention of type 2 diabetes: integrative public health and primary care opportunities, challenges and strategies. Family Practice 29 (Suppl 1i2), i13–i23. 10.1093/fampra/cmr126. 22399542 PMC4705310

[ref15] Haschar-Noé N and Salaméro É (2016) La fabrication d’un contrat local de santé «expérimental». Négociations et compromis sous tensions. [The manufacture of a “experimental” local health contract. Negotiations and compromises under strain.]. Sciences Sociales et Santé 34, 81–105. [in French]

[ref16] Haschar-Noé N , Salamero E and Honta M (2015) La gouvernance différenciée des contrats locaux de santé. [Differentiated governance of local health contracts]. Journal de Gestion et D’économie Médicales 33, 375–388. [in French]

[ref17] Hone T , Macinko J and Millett C (2018) Revisiting Alma-Ata: what is the role of primary health care in achieving the Sustainable Development Goals? The Lancet 392, 1461–1472. 10.1016/S0140-6736(18)31829-4 30343860

[ref18] Honta M , Haschar-Noé N and Salaméro É (2018) L’État à l’épreuve de la régulation territoriale. La mise en négociations des contrats locaux de santé. [The State in the test of territorial regulation. Negotiation of local health contracts]. Négociations 1, 143–155. [in French]

[ref19] Jabot F and Laurent A (2018) Les contrats locaux de santé en quête de sens. [Local health contracts in search of meaning]. Sante Publique France 30, 155–156. [in French] 10.3917/spub.182.0155 30148302

[ref20] Kringos D , Boerma W , Bourgueil Y , Cartier T , Dedeu T , Hasvold T , Hutchinson A , Lember M , Oleszczyk M and Pavlic DR (2013) The strength of primary care in Europe: an international comparative study. British Journal of General Practice 63, e742–e750. 10.3399/bjgp13X674422 PMC3809427 24267857

[ref21] Lebrun LA , Shi L , Chowdhury J , Sripipatana A , Zhu J , Sharma R , Hayashi AS , Daly CA , Tomoyasu N , Nair S and Ngo-Metzger Q (2012) Primary care and public health activities in select U.S. health centers: documenting successes, barriers, and lessons learned. American Journal of Preventive Medicine 42, S191–S202. 10.1016/j.amepre.2012.03.011. 22704437

[ref22] Levesque J-F , Breton M , Senn N , Levesque P , Bergeron P and Roy DA (2013) The interaction of public health and primary care: functional roles and organizational models that bridge individual and population perspectives. Public Health Reviews 35, 14 10.1007/BF03391699.

[ref23] Lionis C , Petelos E , Papadakis S , Tsiligianni I , Anastasaki M , Angelaki A , Bertsias A , Mechili EA , Papadakaki M , Sifaki-Pistolla D and Symvoulakis E (2018) Towards evidence-informed integration of public health and primary health care: experiences from Crete. Public Health Panorama 4, 699–714.

[ref24] LOI n° 2009-879 du 21 juillet 2009 portant réforme de l’hôpital et relative aux patients, à la santé et aux territoires [Reform of hospital, patients, health and territories] (2009) Retrieved from https://www.legifrance.gouv.fr/affichTexte.do?cidTexte=JORFTEXT000020879475&categorieLien=id. [in French]

[ref25] Ministère des Solidarités et de la Santé (2019a) Ma santé 2022 : un engagement collectif. [My Health 2022: ACollective Commitment.] *Ministère des Solidarités et de la Santé* Retrieved 16 May 2019 from https://solidarites-sante.gouv.fr/systeme-de-sante-et-medico-social/ma-sante-2022-un-engagement-collectif/ [in French]

[ref26] Ministère des Solidarités et de la Santé (2019b) Accès aux soins : Le guide pratique pour les élus, [Access to Care: A Practical Guide for Elected Officials]. Retrieved 5 February 2020 from https://solidarites-sante.gouv.fr/actualites/actualites-du-ministere/article/acces-aux-soins-le-guide-pratique-pour-les-elus. [in French]

[ref27] Pratt R , Gyllstrom B , Gearin K , Hahn D , VanRaemdonck L , Peterson K and Baldwin L-M (2017) Primary care and public health perspectives on integration at the local level: a multi-state study. Journal of the American Board of Family Medicine 30, 601–607. 10.3122/jabfm.2017.05.170034. 28923812

[ref28] Pratt R , Gyllstrom B , Gearin K , Lange C , Hahn D , Baldwin L-M , VanRaemdonck L , Nease D and Zahner S (2018) Identifying barriers to collaboration between primary care and public health: experiences at the local level. Public Health Reports 133, 311–317. 29614236 10.1177/0033354918764391 PMC5958390

[ref29] Safon M-O (2018) La loi de modernisation de notre système de santé. [The Act to Modernize Our Health Care System]. 107 p. Retrieved from https://solidarites-sante.gouv.fr/systeme-de-sante-et-medico-social/loi-de-modernisation-de-notre-systeme-de-sante/ [in French]

[ref30] Salaméro É and Haschar-Noé N (2017) Variabilité des formes de gouvernance d’un contrat local de santé: ajustement en situation et légitimation négociée. [Variability in the forms of governance of a local health contract: situation adjustment and negotiated legitimacy.]. Terrains travaux 1, 163–184 [in French].

[ref31] Starfield B , Shi L and Macinko J (2005) Contribution of primary care to health systems and health. The Milbank Quarterly 83, 457–502. 10.1111/j.1468-0009.2005.00409.x. 16202000 PMC2690145

[ref32] Valaitis RK and Canadian Foundation for Healthcare Improvement (2013) Strengthening primary health care through primary care and public health collaboration final report for CFHI. Ottawa, ON: Canadian Foundation for Healthcare Improvement Retrieved 16 December 2018 from http://ra.ocls.ca/ra/login.aspx?inst=centennial&url=https://www.deslibris.ca/ID/235688

[ref33] WHO The 10 Essential Public Health Operations (2019) Retrieved 16 December 2019 from http://www.euro.who.int/en/health-topics/Health-systems/public-health-services/policy/the-10-essential-public-health-operations

[ref35] WHO (2016) Global strategy on human resources for health: Workforce 2030. Retrieved 17 December 2019 from https://apps.who.int/iris/bitstream/handle/10665/250368/9789241511131-eng.pdf;jsessionid=770E7035D98DE33878FCE41EBEF73DB0?sequence=1

[ref34] WHO (2018) Declaration of Astana.

[ref36] WHO Regional Office for Europe (2019) *A vision for Primary Health Care in the 21st century* Retrieved 5 March 2019 from https://www.who.int/docs/default-source/primary-health/vision.pdf

[ref37] Winslow CE (1920) The untilled fields of public health. Science 51, 23–33. 10.1126/science.51.1306.23. 17838891

[ref38] WONCA Working Party on the environment, The planetary health alliance, The clinicians for plenetary health (2019) Declaration calling for family doctors of the world to act on plenetary health. Retrieved 17 December 2019 from https://www.wonca.net/site/DefaultSite/filesystem/documents/Groups/Environment/2019%20Planetary%20health.pdf

